# Betulinic acid induces autophagy-dependent apoptosis via Bmi-1/ROS/AMPK-mTOR-ULK1 axis in human bladder cancer cells

**DOI:** 10.18632/aging.203441

**Published:** 2021-09-12

**Authors:** Yan Zhang, Ning He, Xuejian Zhou, Feifan Wang, Hairong Cai, Shih Han Huang, Xianwu Chen, Zhenghui Hu, Xiaodong Jin

**Affiliations:** 1 Department of Urology, The First Affiliated Hospital, Zhejiang University School of Medicine, Hangzhou 310003, Zhejiang, P.R. China

**Keywords:** betulinic acid, bladder cancer, AMPK pathway, autophagy, apoptosis

## Abstract

Betulinic acid (BA), a pentacyclic triterpenoid isolated from tree bark, exhibits antitumor effects against solid malignancies and triggers autophagy and/or apoptosis in human cancer cells. Nonetheless, the relationship between autophagy and apoptosis and the potential modulatory actions of BA on autophagy-dependent bladder cancer cell death remain unclear. The present study showed that BA exposure significantly suppressed viability, proliferation, and migration of EJ and T24 human bladder cancer cells. These effects reflected caspase 3-mediated apoptosis and could be attenuated or abolished by inhibiting ROS production with N-acetyl-L-cysteine, inhibiting autophagy with chloroquine, or silencing ATG7 with targeted siRNA. BA-induced autophagy was evidenced by epifluorescence imaging of lentivirus-induced expression of mCherry-GFP-LC3B and increased expression of two autophagy-related proteins, LC3B-II and TEM. Moreover, enhanced AMPK phosphorylation and decreased mTOR and ULK-1 phosphorylation suggested BA activates autophagy via the AMPK/mTOR/ULK1 pathway. Accordingly, exposure to dorsomorphin (Compound C), an AMPK inhibitor, and AICAR, an AMPK activator, respectively inhibited and stimulated BA-induced autophagy in EJ and T24 cells. The effects of Bmi-1 overexpression *in vitro* and decreased Bmi-1 expression in BA-treated T24 cell xenografts in nude mice suggested that downregulation of Bmi-1 is the underlying mechanism in BA-mediated, autophagy-dependent apoptosis.

## INTRODUCTION

Bladder cancer is the 2^nd^ commonest malignancy of the urinary system and the 10^th^ most frequent cancer globally [1]. The global cancer report by the International Agency for Research on Cancer estimated that 549,393 new bladder cancer cases and 199,922 deaths from bladder cancer occurred in 2018 [1]. Bladder cancer is mainly treated with surgery combined with postoperative bladder perfusion chemotherapy. However, up to 50% of patients with non-muscle invasive bladder cancer relapse, whereas 15%-20% of those progress to muscle invasive bladder cancer, characterized by a high propensity for spread to other organs [2, 3]. Thus, there is a critical need of new bladder cancer treatments.

Betulinic acid (BA) is a natural pentacyclic triterpene found in the white birch tree (*Triphyophyllum peltatum*) as well as in the jujube tree (*Ziziphus jujuba*) [4, 5]. Recent evidence indicates that BA has several properties useful for the treatment of metabolic disorders, infectious diseases, and cardiovascular and neurological disorders [6]. BA induces cell death, autophagy, and apoptosis in various cancer and non-cancer human cell lines [7–9]. BA also suppresses human multiple myeloma growth by triggering reactive oxygen species (ROS) overproduction [10].

Autophagy, first described by Ashford and Porter in 1962, is a unique self-protection mechanism and a regulator of intracellular homeostasis in eukaryotic cells [11]. In normal circumstances, autophagy ensures cell survival by regulating physiological cellular processes like proliferation, differentiation, senescence, cell death, and defense against pathogens [12]. In cancer, autophagy can be neutral, tumor-promoting, or tumor-inhibiting, depending on the cellular context. Autophagy involves 4 stages: 1) autophagic phagosome formation, 2) autophagosome extension, 3) autophagosome maturation into autophagolysosomes, and 4) autophagy execution [13]. Autophagy can be initiated by activation of AMPK, a central cellular energy sensor, in response to cellular stressors including oxidative stress. The latter is characterized by high production of byproducts of cellular redox reactions, collectively termed reactive oxygen species (ROS). These consist mostly of short-lived, oxygen-containing metabolites with high reactivity and low molecular weight [14, 15]. Nutrient deficiency, ischemia, hypoxia, and reperfusion injury alter homeostasis, reducing the cell’s antioxidant capacity and resulting in excessive ROS generation. ROS accumulation causes oxidative damage, which leads to mitochondrial dysfunction, may precipitate autophagy, and eventually triggers cell death [16].

Polycomb complex protein Bmi-1 was demonstrated to be a prognostic marker in bladder cancer [17]. Knockdown of Bmi-1 in bladder cancer cells was shown to inhibit stemness properties and tumorigenicity of side population cells and to induce apoptosis [18, 19]. Using *in vitro* and *in vivo* approaches, in this study we evaluated the effects of BA on human bladder cancer cell proliferation and migration and examined the role of ROS, the AMPK-mTOR-ULK1 cascade, and Bmi-1 in autophagy-dependent apoptosis triggered by BA exposure. By unmasking the molecular mechanism by which BA restricts progression of human bladder carcinoma cells, our findings may be valuable to advance novel, much needed therapies for bladder cancer patients.

## RESULTS

### BA reduces viability, proliferation, and migration of human bladder cancer cells

**Figure 1:**
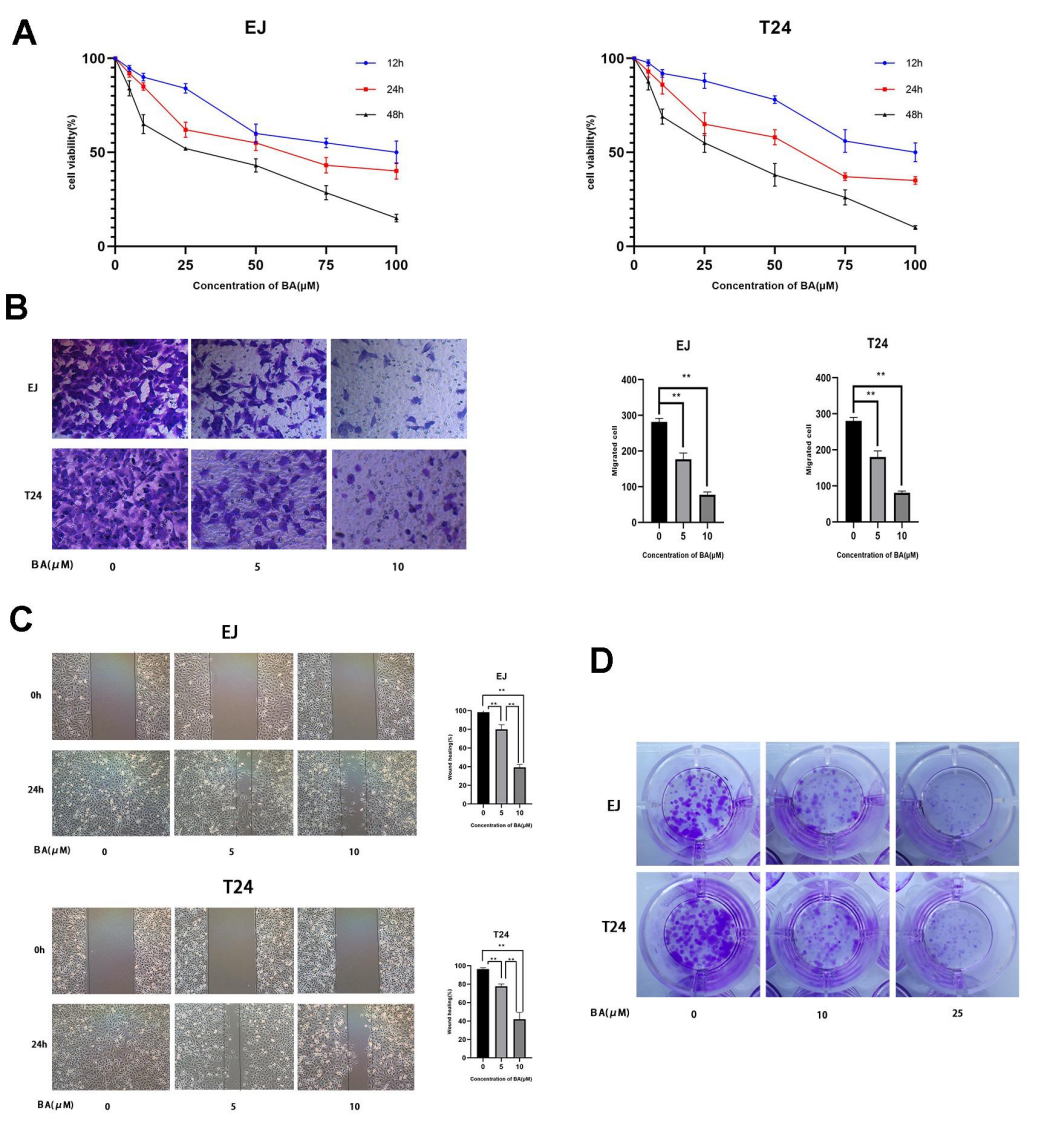
**BA represses viability, proliferation, and migration of human bladder cancer cells.** (**A**) EJ and T24 cells were exposed to BA at specified doses for 24 h and cell viability examined with the CCK-8 assay. (**B**) Transwell migration assay results for EJ and T24 cells. (**C**) Wound healing assay results. (**D**) Colony formation assay results. * *p*<0.05, **p<0.01.

CCK-8-based viability analysis of EJ and T24 human bladder cancer cell lines exposed to BA for 24 h revealed a dose-dependent decrease in cell viability and proliferation (Figure 1A). Moreover, Transwell, wound-healing, and colony formation assays showed that BA exposure significantly suppressed migration and colony formation potential in both cell types (Figure 1B–1D).

### BA triggers caspase-dependent apoptosis in human bladder cancer cells

**Figure 2:**
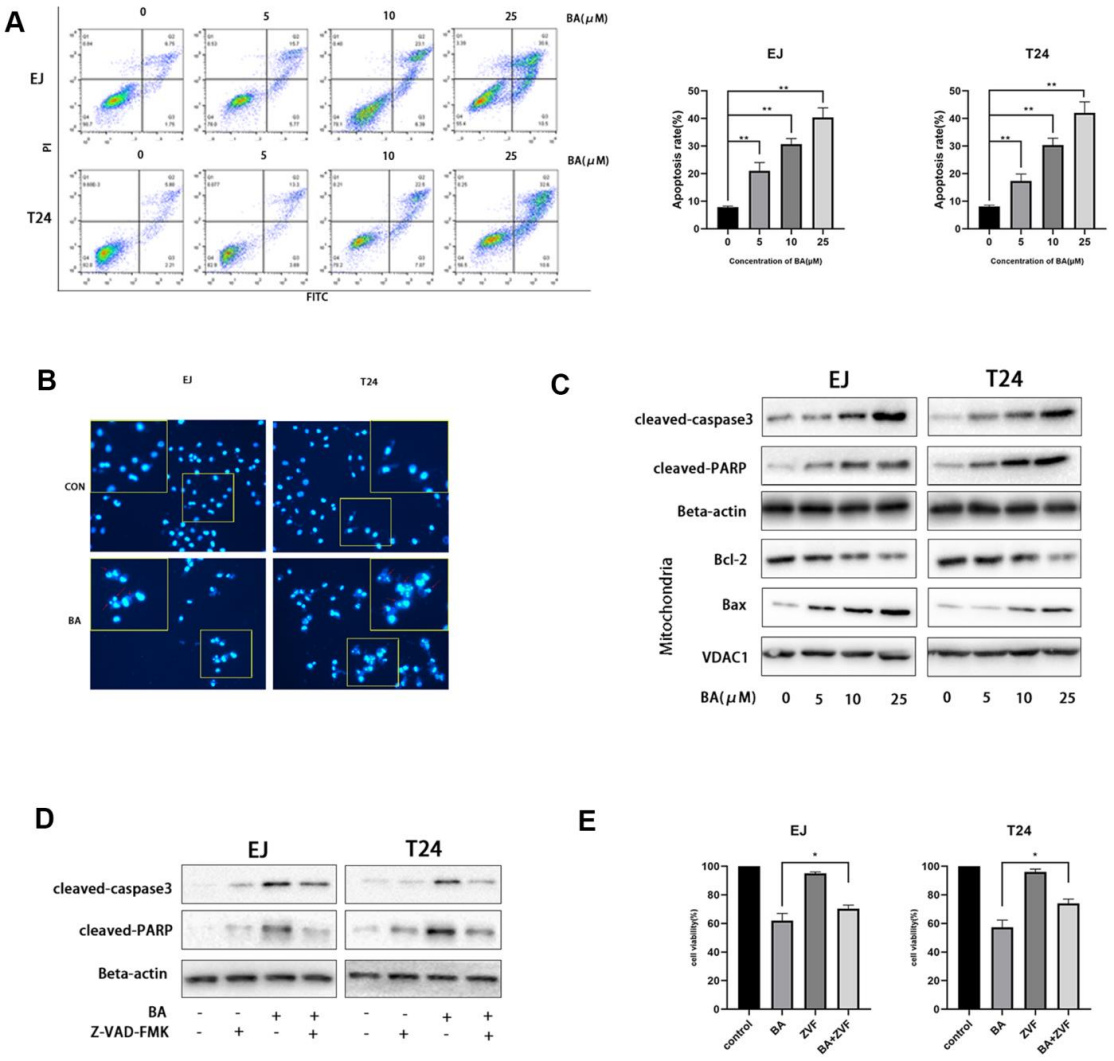
**BA triggers caspase-dependent apoptosis in human bladder cancer cells.** (**A**) Flow cytometric analysis of apoptosis in EJ and T24 cells exposed to BA at various doses. Results are mean ± SD of 3 independent replicates. (**B**) Hoechst 33342 staining of EJ and T24 cells exposed to vehicle (DMSO) or 25 μM BA for 24 h. Representative images show abnormal or apoptotic cells (red arrows). (**C**) Western blot assessment of apoptosis-related factors (Bax, Bcl-2, cleaved PARP, and cleaved caspase-3) in control and BA-exposed cells. β-actin served as loading control. (**D**, **E**) Western blot assessment of cleaved caspase-3 and cleaved PARP expression (**D**) and CCK-8 analysis of cell viability (**E**) in EJ and T24 cells exposed to 25 μM BA with or without 20 μM Z-VAD-FMK (ZVF) for 24 h. con: control, *p<0.05, **p<0.01.

Next, we examined whether BA suppressed cell proliferation by inducing apoptosis. To this end, EJ and T24 cells were exposed to different BA doses, stained with Annexin V-FITC/PI, and apoptosis rates determined by flow cytometry (Figure 2A). This analysis revealed that BA triggered apoptosis dose-dependently. Analysis of nuclear morphology by Hoechst 33342 staining confirmed that BA triggered apoptosis in both cell lines, as evidenced by condensed/fragmented nuclei (Figure 2B). In turn, western blot analysis revealed that the expression of pro-apoptotic factors, namely Bax, cleaved caspase-3, and cleaved-PARP, was dose-dependently stimulated by BA, while levels of Bcl-2, an anti-apoptotic factor, were instead reduced (Figure 2C). To determine whether caspase activation was required for BA-triggered apoptosis, caspase-3 activation was inhibited by co-application of Z-VAD(OMe)-FMK (ZVF). Western blotting assays revealed remarkably reduced cleaved caspase-3, as well as cleaved PARP contents, in ZVF-treated, BA-exposed cells (Figure 2D). In line with these results, CCK-8 assays demonstrated that BA-mediated growth inhibition was attenuated by co-treatment with ZVF (Figure 2E). These results indicate that caspase 3 activation mediates BA-triggered apoptosis in bladder cancer cells.

### BA stimulates autophagy in human bladder cancer cells

**Figure 3:**
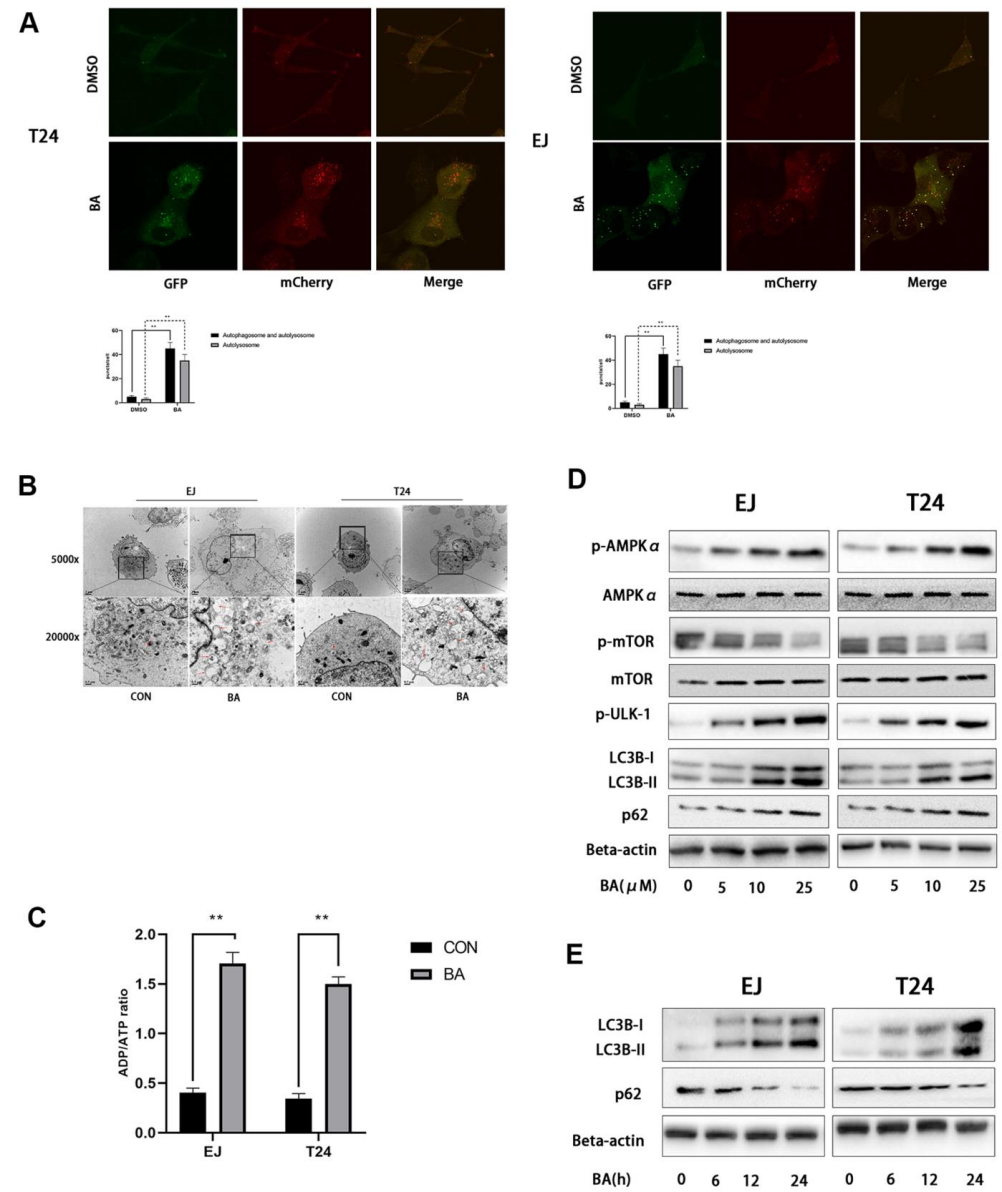
**BA induces autophagy in human bladder cancer cells.** (**A**) Confocal fluorescence microscopy of EJ and T24 cells transfected with mCherry-GFP-LC3B lentivirus and exposed to 25 μM BA for 24 h. (**B**) Transmission electron microscopy (magnification: 5000× and 20,000×) images of EJ and T24 cells exposed to 25 μM BA for 24 h. Arrows indicate autophagic vesicles. (**C**) Luminescent determination of ADP/ATP ratio in EJ and T24 cells exposed to DMSO (control) or BA for 24 h. (**D**, **E**) Western blot analysis of changes in AMPK-mTOR-ULK1 phosphorylation status and p62 and LC3B-II expression upon treatment with the specified BA doses. *p<0.05, **p<0.01.

Since numerous studies indicate that BA activates autophagy in cancer cell lines, we evaluated autophagosome formation in bladder cancer cells transfected with an mCherry-GFP-LC3B lentiviral construct. Under epifluorescence microscopy, red/green (R+G+; i.e. yellow) and red (R+G-) puncta denote, respectively, autophagosome and autophagolysosome formation and are indicative of the overall autophagy level within cells. As shown in Figure 3A, the appearance of both yellow and red puncta was significantly stimulated upon BA treatment compared to control, DMSO-treated cells. Accordingly, on transmission electron microscopy (TEM) analysis, the presence of double-membrane vesicles containing high electron density contents confirmed that BA exposure significantly increased autophagosome formation in both EJ and T24 cells (Figure 3B). Further proof of BA-induced autophagy was obtained by western blot analysis, which revealed that BA treatment dose- and time-dependently increased the expression of the autophagy marker LC3B-II and decreased expression was observed for p62 (Figure 3D, 3E).

### BA activates AMPK/mTOR/ULK1 signaling in human bladder cancer cells

As an important energy sensor, AMPK is activated by adverse stimuli to regulate cell energy metabolism. This is achieved in part by induction of autophagy via the AMPK-mTOR-ULK1 signaling pathway [20, 21]. To determine whether BA activates autophagy in bladder cancer cells via the AMPK-mTOR-ULK1 pathway, we first used a luminescence-based assay to evaluate potential changes in the cellular ADP/ATP ratio. Results showed that BA induced a significant increased in intracellular ADP/ATP ratio in EJ and T24 cells (Figure 3C). In turn, western blot assays showed that BA treatment upregulated p-AMPKα (Thr172) and downregulated both p-mTOR (Ser2448) and p-ULK1 (Ser555) expression dose-dependently in both cell lines (Figure 3D, 3E). These findings suggest that BA induces autophagy in EJ and T24 cells via AMPK-mTOR-ULK1 signaling.

### BA-mediated ROS production contributes to caspase-dependent apoptosis and autophagy

**Figure 4:**
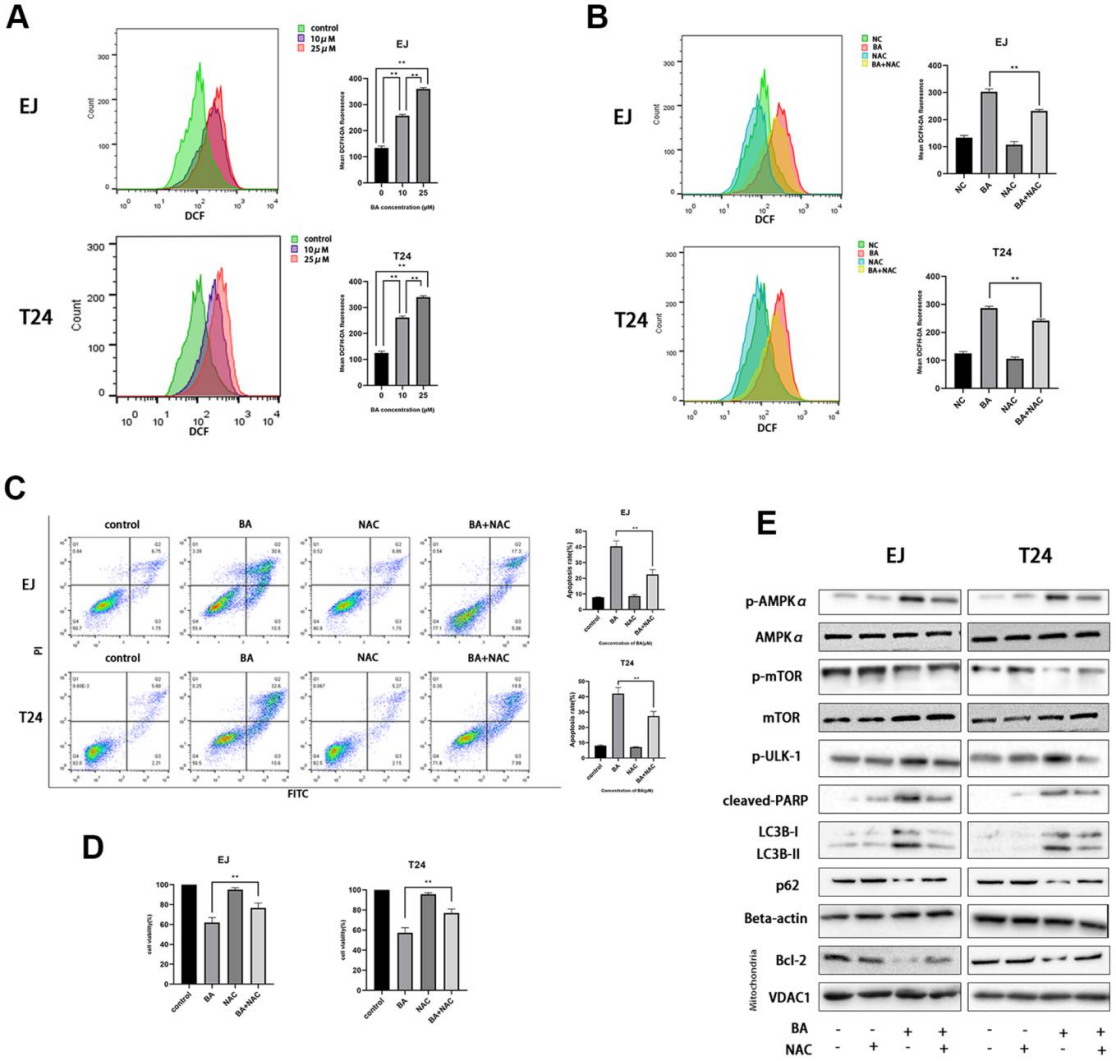
**BA-mediated ROS generation induces caspase-dependent apoptosis and autophagy in bladder cancer cells.** (**A**) Flow cytometry analysis of ROS generation in DCFH-DA-loaded EJ and T24 cells exposed to BA at 0, 10, and 25 μM for 24 h. (**B**) Flow cytometry analysis of ROS generation in EJ and T24 cells pre-exposed to NAC. (**C**) Flow cytometry analysis of apoptosis. (**D**) The CCK-8 assay was employed to evaluate NAC effect on BA-triggered cytotoxicity. (**E**) Western blot analysis of p-AMPKα (Thr172), cleaved PARP, p-mTOR (Ser2448), p-ULK1 (Ser555), Bcl-2, p62, and LC3B II levels. *p<0.05, **p<0.01.

A previous study showed that excess ROS production modulates AMPK and induces autophagy [22]. Flow cytometry analysis of EJ and T24 cells loaded with the ROS indicator DCFH-DA revealed that BA induced ROS production dose-dependently (Figure 4A). Next, we used NAC, an active oxygen scavenger, to assess the role of ROS on BA’s cellular effects. Co-incubation with NAC effectively blocked ROS production (Figure 4B), rescued cell viability, (Figure 4C), and inhibited apoptosis (Figure 4D) induced by BA. Western blot data further illustrated that NAC co-exposure restored basal expression of p-AMPKα (Thr172), p-ULK1, cleaved PARP, and LC3B II and suppressed the expression of p-mTOR (Ser2448), p62 and Bcl-2 (Figure 4E). This suggests that ROS overproduction contributes to BA-triggered apoptosis and autophagy in EJ and T24 cells.

### Autophagy promotes BA-mediated apoptosis

**Figure 5:**
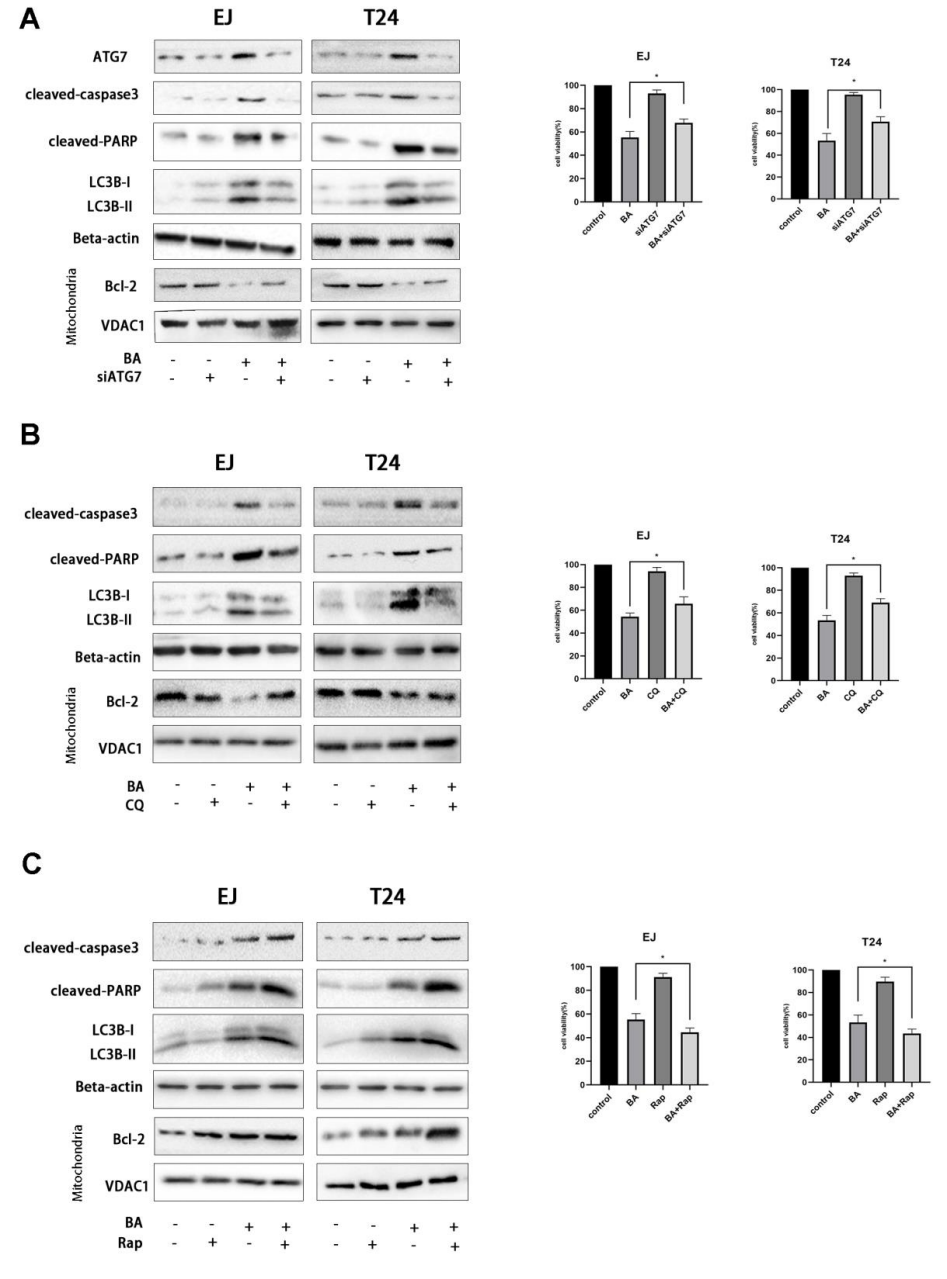
**Autophagy mediates BA-induced apoptosis in bladder cancer cells.** Cell viability and western blot assay results for EJ and T24 cells transfected with siATG7 or control siRNA (**A**) or pre-exposed to 10 μM CQ (**B**) or 250 nM rapamycin (Rap) (**C**) for 4 h prior to incubation with DMSO (control) or 25 μM BA for 24 h. The CCK-8 assay was used to assess cell viability. Western blot was employed to corroborate ATG7 deletion (**A**) and to analyze apoptosis (Bcl-2; cleaved caspase-3; cleaved PARP), and autophagy (LC3B-II) markers (**A**–**C**). *p<0.05, **p<0.01.

**Figure 6:**
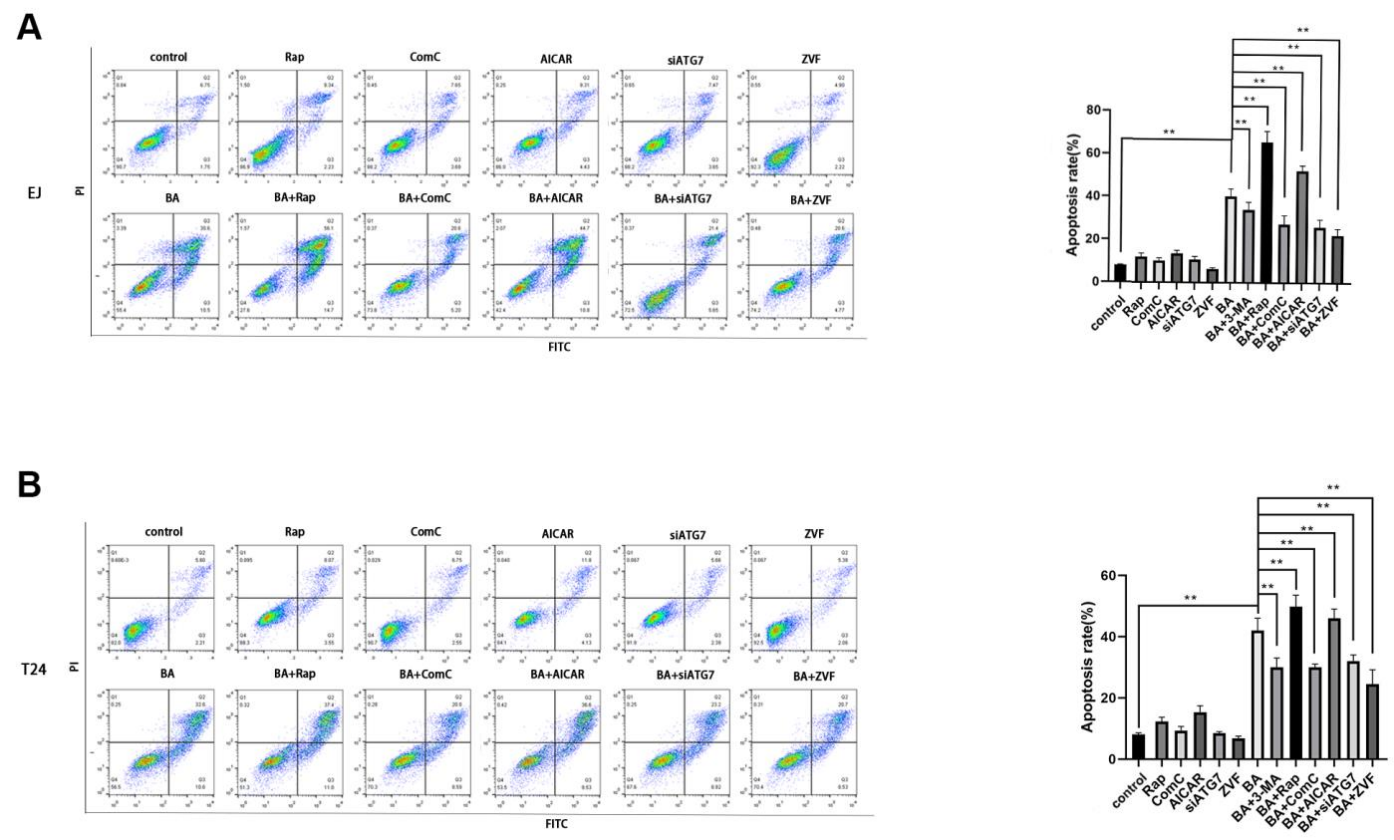
**Autophagy inhibition blunts BA-mediated apoptosis.** Flow cytometry analysis of apoptosis in EJ cells (**A**) and T24 cells (**B**) treated with different autophagy-modulating agents. *p<0.05, **p<0.01.

To explore the potential link between BA-mediated autophagy and apoptosis, ATG7-targeted siRNA (siATG7) and chloroquine (CQ) were alternatively employed to suppress autophagy in EJ and T24 cells. ATG7 interacts with LC3B to promote early autophagosome formation, while CQ inhibits autophagy by impairing autophagosome-lysosome fusion [23]. Western botting data confirmed that siATG7 significantly reduced ATG7 expression in both cell types (Figure 5A). As shown in Figure 5A, 5B, both CQ and siATG7 remarkably rescued cell viability in BA-treated cells. In turn, western blot assays of apoptosis-related factors (cleaved PARP, cleaved caspase 3, and Bcl-2) and autophagy-associated proteins (ATG7 and LC3B-II) showed that BA-induced apoptosis and autophagy were both attenuated after treatment with siATG7 or CQ (Figure 5A, 5B). Consistent with western blot findings, Annexin V flow cytometry assays showed that transfection with siATG7 partially inhibited apoptosis in both cell types (Figure 6). For further verification, we explored the effect of rapamycin (Rap), which activates autophagy by inhibiting mTOR, on BA-treated cells. Further indicating that BA triggers autophagy-dependent apoptosis, Rap exposure counteracted the inhibitory effects of siATG7 and CQ on BA-induced autophagy and apoptosis (Figures 5C, 6).

### AMPK-mTOR-ULK1 signaling is critical for BA-triggered, autophagy-dependent apoptosis

**Figure 7:**
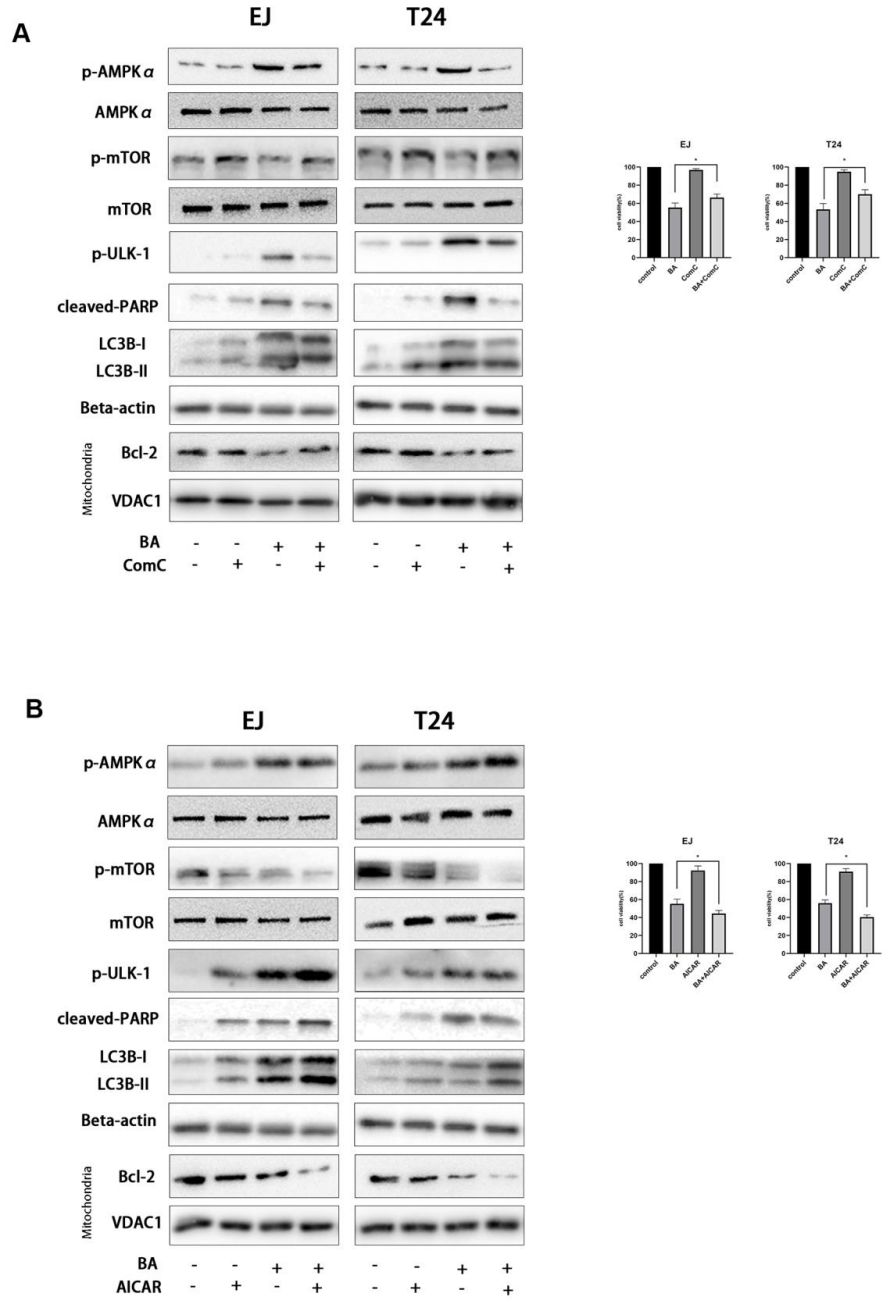
**BA-triggered autophagy-dependent apoptosis requires activation of the AMPK-mTOR-ULK1 axis.** Cell viability and western blot assay results for EJ and T24 cells pre-exposed to 10 μM dorsomorphin (ComC) (**A**) or 500 μM AICAR (**B**) for 4 h and incubated with DMSO (control) or 25 μM BA for another 24 h. The CCK-8 assay was used for analysis of cell viability. Western blot was employed to determine the expression of cleaved PARP, p-AMPKα (Thr172), p-mTOR (Ser2448), p-ULK1 (Ser555), Bcl-2, cleaved caspase-3, and LC3B-II. *p<0.05, **p<0.01.

To further clarify the involvement of the AMPK/mTOR/ULK1 signaling cascade in BA-triggered autophagy and apoptosis, we treated EJ and T24 cells with dorsomorphin (Compound C; ComC), an AMPK inhibitor, or with AICAR, an AMPK activator. Notably, addition of ComC rescued cell viability and attenuated or abolished the changes in the expression of apoptosis and autophagy markers elicited by BA (Figure 7A). Conversely, the addition of AICAR further decreased cell viability and potentiated the pro-autophagic and pro-apoptotic effects of BA (Figure 7B). Consistent with these findings, flow cytometry data showed that ComC and AICAR respectively inhibited and stimulated apoptosis in BA-treated cells (Figure 6). These results strongly suggest that BA acts through AMPK to induce autophagy and apoptosis in bladder cancer cells.

### Bmi-1 overexpression partially inhibits BA-mediated ROS overproduction, cell migration, and autophagy-dependent apoptosis in bladder cancer cells

**Figure 8:**
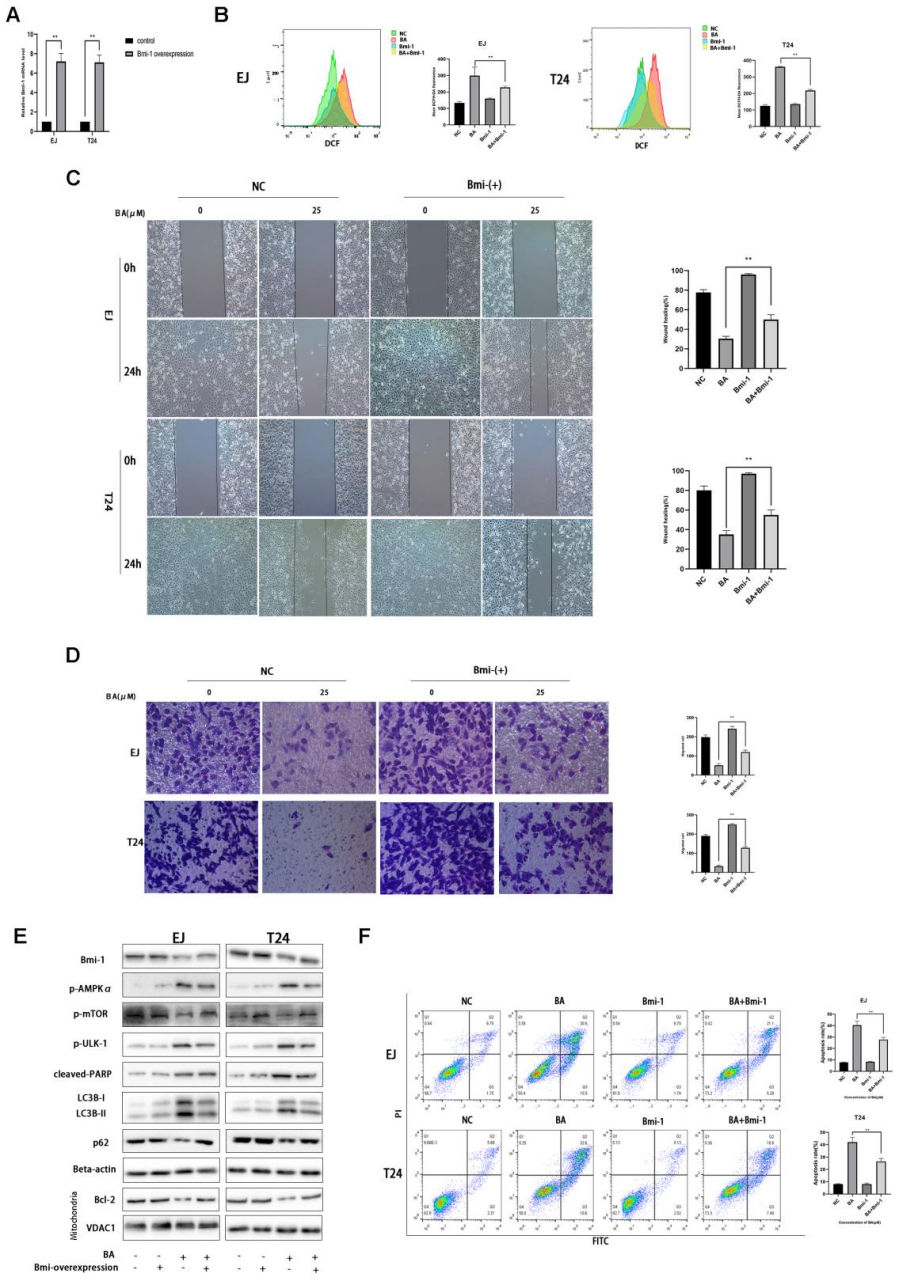
**Bmi-1 overexpression partially reverses BA-induced ROS overproduction, migration inhibition, and autophagy-dependent apoptosis.** Stable overexpression of Bmi-1 was established in EJ and T24 cells (EJ-con/EJ-Bmi-1 and T24-con/T24-Bmi-1) via lentiviral transduction before exposure to BA. (**A**) RT-qPCR analysis of relative Bmi-1 expression in control and Bmi-1-transduced cells. (**B**) Assessment of intracellular ROS contents. (**C**) Wound healing assay results. (**D**) Transwell migration analysis. (**E**) Western blot analysis of p-AMPKα, p-mTOR, p-ULK1, Bcl-2, cleaved caspase-3, Bmi-1, LC3B-II, p62, and cleaved PARP expression. (**F**) Apoptosis determination by flow cytometry. *p<0.05, **p<0.01.

Along with other studies, we have previously shown that Bmi-1, an oncogene overexpressed in various human cancers in relation with enhanced proliferation and metastasis, has a vital role in autophagy and ROS generation [24–26]. Western blot assays showed that BA exposure downregulated Bim-1 (8E). To assess whether Bmi-1 expression counteracts BA-induced ROS production, migration inhibition, and autophagy induction in bladder cancer cells, we overexpressed Bmi-1 via lentiviral transfection. After confirming Bmi-1 overexpression by RT-qPCR (Figure 8A), cellular ROS and migration assays showed that Bmi-1 overexpression suppressed or attenuated BA-mediated ROS production (Figure 8B) and migration potential (Figure 8C, 8D) in both cell types. Furthermore, Bmi-1 overexpression also attenuated or abrogated the changes in the expression of apoptosis (cleaved PARP, Bcl-2, and cleaved caspase 3) and autophagy (LC3B-II) markers, as well as the alterations in the phosphorylation status of AMPK-mTOR-ULK1 proteins elicited by BA (Figure 8E). In turn, flow cytometry and cell viability analyses also confirmed that Bmi-1 overexpression prevented apoptosis and rescued viability in BA-treated EJ and T24 cells (Figure 8F). These data indicate that the anticancer effects of BA can be abrogated by overexpression of Bmi-1.

### BA represses bladder cancer cell growth *in vivo*

**Figure 9:**
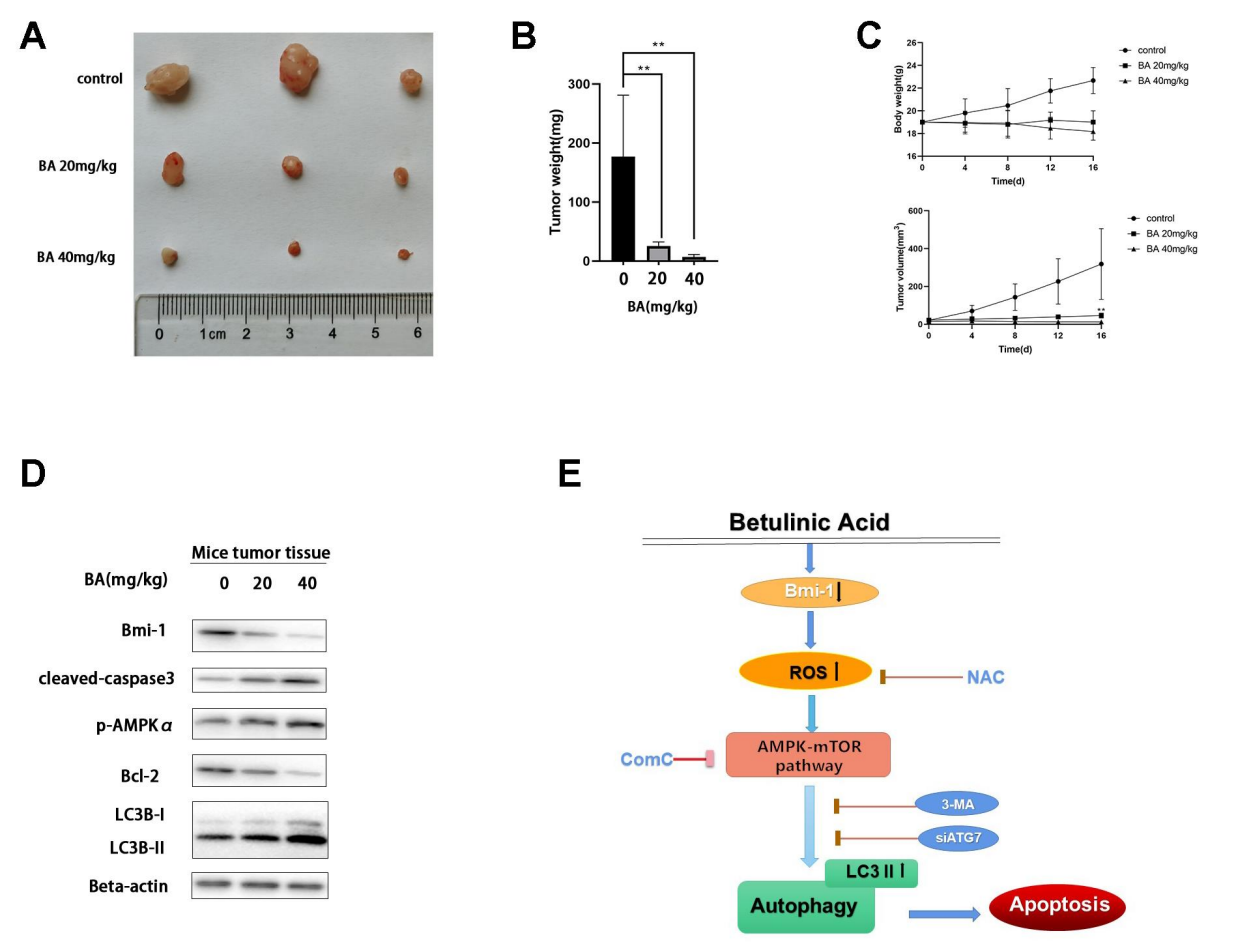
**BA represses T24 cell tumor xenograft growth *in vivo*.** (**A**, **B**) Tumor volume measurements. (**C**) Tumor weight measurements. (**D**) Western blot assessment of Bmi-1, cleaved caspase-3, p-AMPKα, Bcl-2, and LC3B-II expression in excised xenograft tumor tissues. (**E**) Schematic of this study results.

To evaluate whether BA can inhibit bladder cancer cell growth *in vivo*, T24 cells were subcutaneously injected into the flanks of immunocompromised mice. When tumors became palpable, saline (control) or BA (20 or 40 mg/kg) were administered daily for 16 days until sacrifice. As shown in Figure 9A, 9B, BA treatment significantly suppressed tumor volume and weight. Additionally, western blot analyses indicated Bmi-1 downregulation and significant upregulation of cleaved caspase 3 and LC3B-II, as well as p-AMPK, in tumors excised from BA-treated mice (Figure 9D).

## DISCUSSION

Current treatments for bladder cancer involve chemotherapy, radiotherapy, immunotherapy, and surgery [27]. However, successful treatment is limited by long treatment cycles and high recurrence and progression rates. Thus, novel effective therapies are urgently needed. In this study, we provide evidence of the inhibitory effects of BA, a natural compound isolated from several tree species, against bladder cancer growth and migration *in vitro* and *in vivo*, and provide an account of the underlying mechanisms.

Apoptosis, a tightly regulated form of cell death discovered in the 1970s [28], is characterized by changes in cell morphology, including nuclear shrinkage (pyknosis), chromatin fragmentation, and cytoplasmic condensation [29]. Caspases (cysteine-aspartic proteases) are pivotal modulators of apoptosis. Once activated, initiator caspases 2, 8, 9, and 10 activate downstream effector caspases 3, 6, and 7. Among these, caspase 3 is a key executioner of apoptosis [30]. In this study, we report that BA inhibits bladder cancer cell progression and activates apoptosis by upregulating cleaved caspase 3, Bax, and cleaved PARP and by downregulating the anti-apoptotic factor Bcl-2. Accordingly, combined treatment with BA and Z-VAD(OMe)-FMK, a caspase 3 inhibitor, dramatically rescued cells from BA-induced apoptosis.

Autophagy is commonly exploited by cancer cells to fulfill the high metabolic demands of tumors. However, the effects of autophagy on cancer cell progression are context-dependent and may in some cases restrict growth [31]. Hence, modulation of autophagy represents an enticing anticancer strategy. Alizadeh et al. showed that autophagy stimulates epithelial to mesenchymal transition and thus facilitates migration in lung cancer cells [32]. In contrast, Jin et al. reported that BECN1-dependent autophagy inhibits bladder cancer cell growth [33]. Conversion of LC3-I to LC3-II is a hallmark of autophagy initiation. Here, we found that BA exposure markedly increased LC3B-II expression in human bladder cancer cells. In turn, increased autophagosome formation upon BA treatment was confirmed by TEM and by confocal microscopy in cells expressing mCherry-GFP-LC3B.

The AMPK/mTOR pathway was shown to modulate autophagy in liver, renal, and heart disease [34–36]. AMPK is essential for autophagy initiation under cellular stress, including glucose starvation and ROS-related oxidative stress [37, 38]. In line with previous studies indicating that BA modulates ROS production in various cell lines [10, 39, 40], we show here that exposure to the ROS scavenger NAC attenuates BA-mediated ROS overproduction and prevents activation of the AMPK-mTOR-ULK1 cascade, resulting in autophagy inhibition.

Multiple studies have reported the intricate relationship between autophagy and apoptosis [41–43]. He et al. demonstrated that members of the Bcl-2 family of anti-apoptotic proteins can interact with Beclin 1 and inhibit autophagy [44]. Using ATG7-targeted siRNA, CQ, and ComC to repress autophagy, and Rap and AICAR to stimulate it, we found that autophagy inhibition and induction suppressed and stimulated, respectively, BA-mediated apoptosis in bladder cancer EJ and T24 cells. Along these lines, a potential role for the Bmi-1 oncogene on BA-mediated autophagy and apoptosis was suggested by *in vitro* experiments that showed that Bmi-1 overexpression abrogated both phenomena in BA-treated cells.

Lastly, we demonstrate that BA administration suppresses bladder cancer xenograft growth in mice. In line with our *in vitro* results, western blot analysis revealed both autophagy induction and extensive apoptosis in tumors from BA-treated mice. Interestingly, concomitant downregulation of the Bmi-1 oncogene and upregulation of p-AMPKα would suggest that suppression of Bmi-1 underlies BA-mediated, AMPK-mTOR-ULK1-dependent, activation of autophagy and apoptosis in tumor cells. While these data are indeed consistent with the pro-apoptotic effects of Bmi-1 knockdown reported in bladder cancer cells [45, 46], additional experiments are clearly needed to ascertain the modulatory influence of BA on Bmi-1 expression and activity, as well as the link between Bmi-1 and both autophagy and apoptosis in bladder cancer cells. To illustrate the viewpoint more clearly, we created a schematic of this study results (Figure 9E).

In conclusion, using *in vitro* and *in vivo* approaches, this study reported the growth-suppressive effect of BA on human bladder cancer cells. Our data suggest that the underlying molecular mechanisms involve autophagy-dependent apoptosis via Bmi-1/ROS/AMPK-mTOR-ULK1 signaling. These findings suggest the therapeutic use of BA as a potential strategy for bladder cancer treatment.

## MATERIALS AND METHODS

### Cell culture and treatments

Human bladder cancer EJ and T24 cells were supplied by the Chinese Academy of Sciences. Cells were cultured in RPMI 1640 (HyClone, USA) enriched with 10% FBS (Gibco) at 37° C with 5% CO_2_ and passaged at 80%-90% confluence. For cell viability and western blot assays, cells were pre-treated with 5 mM NAC (3 h), 10 μM dorsomorphin (ComC; 4 h), 500 μM AICAR (4 h), 10 μM CQ (4 h), or 250 nM rapamycin (Rap; 4 h) and then incubated with 25 μM BA for another 24 h. Z-VAD-FMK (20 μM) was applied for 24 h concomitantly with 25 μM BA. DMSO (v/v, 1:1,000) was used as vehicle control for comparison with BA effects.

### Reagents and antibodies

BA was supplied by MedChemExpress, USA. CCK-8 kit, dorsomorphin, AICAR, CQ, and Rapamycin (Rap) were provided by Selleck Chemicals, USA. Hoechst 33342 was procured from Sigma, USA. RiboBio Co., China, supplied ATG7 siRNA (SIGS0005319-1). NAC (N-acetyl-L-cysteine) was provided by Beyotime Biotechnology, China. Z-VAD-FMK powder was provided by Feifan Wang. The apoptosis detection kit (Cat. 556547) was purchased from BD Biosciences, USA. Antibodies against Bmi-1 (ab126783), Bcl-2 (ab182858), mTOR (ab2732), p-mTOR (Ser 2448), Bax (ab32503), AMPKα (ab32047), and LC3B (ab192890) were purchased from Abcam, UK. Anti-β-actin antibody (20536-1-AP) was supplied by Proteintech, USA. Antibodies against p-ULK1 (Ser555; Cat. 5869), cleaved poly (ADP-ribose) polymerase (PARP) (Cat. 9541), p-AMPKα (Cat. 2535), SQSTM1/p62 (Cat. 8025), and cleaved caspase 3 (Cat. 9664) were procured from Cell Signaling Technology, USA.

### Cell viability assessment

Cells (10,000/well) were inoculated into 96-well plates in 100 μL of culture medium. After pre-treatment with the specified drugs/siRNAs, different concentrations of BA were applied for 24 h. Afterwards, 10 μL CCK-8 reagent was introduced into every well and incubated at 37° C for 4 h. A microplate reader (Bio-Rad, USA) was employed to determine absorbance at 450 nm.

### Wound healing assay

Cells were inoculated on six-well plates and allowed to grow to 80%-90% confluence. Afterwards, pipette tips were employed to scratch a linear wound. Cells were then incubated in serum-free media and exposed to different treatments for 24 h. Imaging was done at 0 and 24 h under light microscopy. The rate of migration was computed with Image J software (NIH, USA).

### Transwell migration assay

Cells were resuspended in serum-free media and loaded onto the upper compartment of Transwell chambers (costar, Corning, USA). The lower compartment contained complete medium as chemoattractant. After 48 h of incubation at 37° C/5% CO_2_, remaining cells in the upper compartment were removed and cells that migrated to the lower surface of the membrane were fixed in 4% PFA for 10 min. After crystal-violet staining, migrating cells were counted at 400× under a microscope.

### Colony formation assay

Cells (1,000 per well) were inoculated into 6-well plates and cultured in fresh medium containing BA or DMSO, with media changes every 3 days. After 12 days, cells were fixed for 10 min in PFA 4% and crystal violet staining was performed at room temperature (RT). The number of colonies with >50 cells was estimated by microscopy.

### Apoptosis assay

After experimental treatments, cultured cells were resuspended and stained with Annexin V-FITC and PI in binding buffer according to the kit’s protocol. The cells were incubated for 15 minutes at RT and apoptosis assessed with a flow cytometer (BD Biosciences).

### Hoechst 33342 analysis

For nuclear morphology analysis, cultured cells were exposed to 0.5 or 1 μM BA for 24 h, stained with Hoechst 33342 for 10 min, rinsed twice with PBS, and observed by fluorescence microscopy.

### Western blot analysis

RIPA buffer supplemented with 1 mM PMSF was employed to lyse the cells. Lysates were cleared through centrifugation at 12,000 RPM for 5 min, followed by protein quantitation using a BCA assay (Beyotime). Protein samples were mixed with 5X loading buffer, heated for 10 min, and 10 μg of protein per sample was fractionated by 12% SDS-PAGE and blotted onto PVDF membranes. Afterwards, 5% dry milk was employed to block the membranes at RT for 1 h before exposure to the specified primary antibodies at 4° C overnight. After three washes in TBS, HRP-labeled secondary antibodies were applied at RT for 1 h before signal development using ECL substrate. Band intensities were then measured on ImageJ. For detection of Bcl2, Bax, and VDAC1, mitochondria were separated using a Cell Mitochondria Isolation kit (Beyotime Biotech, Cat. C3601) and western blot analysis carried out on the extracts.

### Transmission electron microscopy (TEM)

Following experimental treatments, cells were fixed with 2.5% glutaraldehyde containing sodium cacodylate at 4° C for at least 6 hours. The cells were next post-fixed with 1% osmium tetroxide, dehydrated, embedded, and cut into 50 nm-thick sections. After staining with 3% uranyl acetate and lead citrate, cellular ultrastructure was examined by TEM (H-7650; Hitachi, Japan).

### Autophagosome detection

Cells transfected with mCherry-GFP-LC3B lentivirus were inoculated into glass bottom culture dishes and grown overnight before exposure to the specified treatments for 24 h. The cells were then rinsed using PBS and fluorescent puncta counted under confocal microscopy (FV1000, Olympus, Japan).

### ADP/ATP assay

A commercial kit (ADP/ATP Ratio Assay Kit MAK135; Sigma-Aldrich) was used to estimate cellular ADP/ATP ratio according to the manufacturer’s instructions.

### Determination of intracellular ROS content

DCFH-DA (Beyotime) was employed to measure ROS in both EJ and T24 cells. The cells were exposed to BA at 0, 25, or 50 μM, with or without 5 mM NAC, for 24 h. Next, 1×10^6^ cells were collected in 1 mL serum-free medium containing 10 μM DCFH-DA and incubated for 30 min at 37° C in the dark. Cells were then rinsed three times with serum-free medium and intracellular ROS production assayed by flow cytometry.

### RNA interference

Cells were allowed to grow to 30% confluence and then transfected with ATG7 siRNA (RiboBio, China) or control siRNA for 48 h. Culture media were then replaced and transfected cells used for downstream analyses.

### Bmi-1 overexpression and RT-qPCR analysis

Overexpression of Bmi-1 in EJ and T24 cells was achieved by lentiviral transfection with vectors. TRIzol reagent was employed to isolate total cellular RNA, from which cDNA was generated using a reverse transcription kit (Cat. RR037, Takara, Japan). Bmi-1 mRNA levels were determined with a SYBR Green real-time PCR kit (Cat. RR420, Takara) on a QUANT5 PCR system (Applied Biosystems, USA) using GAPDH as reference gene. The following primers were used:

Bmi-1: Forward: TGGATCGGAAAGTAAACAAAGAC,

Reverse: TGCATCACAGTCATTGCTGCT;

GAPDH: Forward: GATATTGTTGCCATCAATGAC,

Reverse: TTGATTTTGGAGGGATCTCG.

### *In vivo* tumorigenesis assay

T24 cells (5 × 10^6^) were implanted subcutaneously into the flanks of 4-week-old, male nude mice. When the tumors reached macroscopic size, the mice were randomly divided into three groups: Control (saline; n = 3); BA 20 mg (20 mg/kg/day, n = 3); and BA 40 mg (40 mg/kg/day, n = 3). Intraperitoneal saline/BA injections were administered daily over 16 days. After 16 days of treatment, the mice were killed and the tumors were removed, weighted, and measured: Total tumor volume (mm^3^) = L × W^2^/2(L = length and W = width).

### Statistical analyses

Data are indicated as mean ± SD. Differences between groups were analyzed on SPSS (IBM, USA) using one- or two-way analysis of variance. *P*<0.05 indicated significance.
